# Efficacy of endoluminal interventional therapy in diabetic peripheral arterial occlusive disease: a retrospective trial

**DOI:** 10.1186/1475-2840-11-17

**Published:** 2012-02-28

**Authors:** Liang Xiao, De-sheng Huang, Jia-jie Tong, Jing Shen

**Affiliations:** 1 Department of Radiology, the First Hospital of China Medical University, 155# Nanjing North Road, Shenyang 110001, Liaoning, People's Republic of China; 2 Department of Mathematics, College of Basic Medical Science, China Medical University, 92# Bei-er Road, Shenyang 110001, Liaoning, People's Republic of China

**Keywords:** Diabetic Angiopathy, Efficacy, Percutaneous Transluminal Angioplasty, Stents

## Abstract

**Background:**

The purpose of this study was to assess the efficacy of interventional therapy for peripheral arterial occlusive disease and the difference between diabetic patients and non-diabetic patients.

**Methods:**

139 consecutive patients between September 2006 and September 2010 who underwent percutaneous lower extremity revascularization for arterial lesions were divided into diabetes group (n = 62) and non-diabetes group (n = 77). Before intervention, rest ankle brachial indexes and three dimensional computed tomography angiography from abdominal aorta to tiptoe were performed. The interventional treatments included angioplasty with or without stenting. The clinical outcomes included rest ankle-brachial indexes, primary patency rates, secondary patency rates and limb-salvage rates for 6-month, 12-month, 24-month and 36-month after treatment. The primary and secondary patency rates of all interventions and the limb-salvage rates of the patients are illustrated by Kaplan-Meier curves and compared by log-rank analysis.

**Results:**

The interventional operation success rates were 98.4% (61/62) in diabetes group and 100% (77/77) in non-diabetes group. The re-interventional operation success rates were 85.7% (18/21) in diabetes group and 76.9% (20/26) in non-diabetes group. The mean value of ankle brachial indexes was significantly increased after intervention (0.397 ± 0.125 versus 0.779 ± 0.137, t = -25.780, *P *< 0.001) in diabetes group and (0.406 ± 0.101 versus 0.786 ± 0.121, t = -37.221, *P *< 0.001) in non-diabetes group. Perioperative 30-day mortality was 0%. Major complications included groin hematoma in 7.2%, and pseudoaneurysm formation 2.2%. In diabetes group, 6, 12, 24, and 36-month primary patency rates were 88.7% ± 4.0%, 62.3% ± 6.6%, 55.3% ± 7.0%, and 46.5% ± 7.5%; secondary patency rates were 93.5% ± 3.1%, 82.3% ± 5.1%, 70.8% ± 6.5%, and 65.7% ± 7%; limb-salvage rates were 95.2% ± 2.7%, 87.7% ± 4.4%, 85.5% ± 4.8%, and 81.9% ± 5.8%. In non-diabetes group, 6, 12, 24, and 36-month primary patency rates were 90.9% ± 3.3%, 71.8% ± 5.4%, 71.8% ± 5.4%, and 60.9% ± 6.2%; secondary patency rates were 96.1% ± 2.2%, 91.6% ± 3.3%, 82.7% ± 4.8%, and 71.8% ± 6.2%; limb-salvage rates were 97.4% ± 1.8%, 94.4% ± 2.7%, 90.6% ± 3.7%, and 83.1% ± 5.4%. The differences between two groups were not significant (*P *> 0.05).

**Conclusion:**

With a low risk of morbidity and mortality, the percutaneous revascularization accepted by patients does not affect ultimate necessary surgical revascularization and consequently should be considered as the preferred therapy for chronic lower extremity ischemia. The efficacy and prognosis of interventional therapy in diabetic patients is similar that in non-diabetic patients.

## Introduction

Diabetes is a common concomitant condition for the patients with peripheral arterial occlusive disease (PAOD) and is increasing in prevalence [1]. Diabetics with PAOD carry a considerably worse prognosis than those without diabetes [2]. They account for more than half in the non-traumatic lower limb amputations. 20% of these patients with infected foot wounds ended up with some type of lower extremity amputation.

Previously, percutaneous lower extremity arterial revascularization was offered only to the patients who had contraindications to open surgical bypass or had used up all surgical options. In recent years, however, an increasing number of patients have undergone percutaneous intervention as a first-line therapy for arterial occlusive disease of the lower extremity [3]. Successful revascularization could reduce the rate of major amputation, improve the healing of foot ulcers and raise the quality of life in diabetic patients with PAOD. Furthermore, an increasing number of techniques for percutaneous therapy have become available, including laser angioplasty, drug eluting balloon, absorbable metal stent and rotational atherectomy [4-8], thereby expanding the range and type of lesions suitable for percutaneous treatment.

In the study we sought to evaluate lower extremity arterial interventional therapy feasibility, revascularization efficacy, complications and limb salvage rate in a population of consecutive diabetic patients and non-diabetic patients hospitalized for PAOD.

## Materials and methods

A retrospective research of the institutional database was performed to identify 68 diabetic patients (diabetes group) and 80 non-diabetic patients (non-diabetes group) between September 2006 and September 2010 who underwent percutaneous revascularization for lower extremity arterial lesions (both stenoses and occlusions). After a complete description of the study to the patients, the written informed consent was obtained in accordance with National Health and Medical Research Council guidelines. The study has been approved by the Ethics Committee of Hospital. Before intervention, rest ankle brachial indexes (ABI) and three dimensional computed tomography angiography (3D-CTA) from abdominal aorta to tiptoe were performed in all patients. Indications for interventional procedure included debilitating claudication or limb-threatening ischemia and lower extremity arterial lesions found by 3D-CTA.

A contralateral femoral approach was used in the majority of patients while an ipsilateral antegrade femoral approach was only employed when isolated tibial arterial disease was anticipated from preoperative studies. Interventional operations were performed through a 5 F or 6 F sheath (length 11-45 cm). Selective angiography was performed to localize lesions and measure the range of lesions by using 4 F or 5 F catheter. The decision to intervene was made after diagnostic angiography. Deciding whether to proceed with revascularization operation was based on the necessity of clinical symptoms instead of the extent of the collateralization. All patients were anticoagulated with intravenous unfraction heparin (5,000 units) before lesion being crossed.

Under monitoring with X-ray, lesions were crossed with either luminal or subintimal technique using hydrophilic guide wires (0.035, 0.018 or 0.014 inch). Guide wires were supported using 4 F or 5 F angled catheter. Reentry into the arterial luminal space beyond the occlusive lesion was confirmed by angiography before further intervention. Percutaneous transluminal angioplasty (PTA) was performed with appropriately sized noncompliant balloons (2.5-8.0 mm diameter) for the treated vessel, with inflation times ranging from 60 to 180 seconds at 6 to 15 atmosphere of pressure. Stent was implanted selectively for > 30% residual stenosis or flow-limiting dissections. Re-examination angiography with evaluation of the extent of residual stenosis and distal runoff was performed after interventions. PTA success was defined as dilation of all arterial lesions with a residual stenosis of ≤20%. Stent technical success was defined as a residual stenosis of < 20% after stent placement. All interventional procedures were performed under local anesthesia.

After arterial intervention, patients received 0.4 ml low molecular heparin injection at twice daily for a week. Activated clotting times were not monitored. Aspirin 100 mg was administered daily to all patients postoperatively unless contraindicated. For patients undergoing stent placement, a loading dose of Clopidogrel 300 mg was administered in ward, followed by 75 mg/d for 1 year. ABI was obtained one week after the procedure.

Patients were evaluated postoperatively and then at 6-month intervals by physical examination (pulses and presence or absence of claudication or rest pain) and by vascular laboratory exam (ABI and arterial duplex ultrasound). Patency was determined primarily by arterial duplex of the treated vessel and secondarily by ABI and clinical parameters. Loss of patency on arterial duplex was defined as the presence of an occlusion or a restenosis associated with a velocity ratio of greater than 4:1(relative to the segment proximal to the treated region). Percutaneous treatment failure was defined as loss of patency by anatomic or hemodynamic measures without effective revascularization. During follow-up a patient was considered to lose patency if restenosis or occlusion was detected in any of the lesions treated.

All analyses were conducted using the Statistical Package for Social Sciences, version 11.5 (SPSS). Continuous variables were described with summary statistics such as means and standard deviations. Categorical variables were described with frequencies and percentages. Student's *t *test and chi square analysis were used as appropriate to evaluate between-group differences in baseline characteristics, and changes of ABI before and after primary intervention. Primary and secondary patency and limb salvage rates were computed by Kaplan-Meier survival curves and compared by log-rank analysis. The difference was considered statistically significant at p < 0.05.

## Results

148 patients were enrolled in the trial. 9 patients were withdrawn from the study because of lost in follow-up. This led to 139 (93.9%) patients who were included in the study. The two groups did not differ significantly in demographic or clinical characteristics and baseline ABI (Table 1).

**Table 1:** Demographic and Clinical Characteristics of 139 patients Across Treatment Groups at Baseline

Characteristics	Diabetes group (n = 62)	Non-diabetes group (n = 77)	Test Statistics	*P* Value
Age(years)	64.7 ± 11.0	65.2 ±9.8	-0.308	0.759
Male	37(59.7%)	49(63.6%)	0.228	0.633
history of tobacco	33(53.2%)	45 (58.4%)	0.379	0.538
hypertension	36(58.1%)	53 (68.8%)	1.729	0.189
coronary artery disease	19(30.6%)	21 (27.3%)	0.191	0.662
Treatment with insulin	45 (72.6%)	0 (0%)	Fisher's exact test	0.000
Pre-operation ABI	0.396 ± 0.125	0.406 ± 0.101	0.113	0.910
**Lesion location**				
Iliac artery	26(23.2%)	32(23.7%)	0.002	0.964
Femoral artery	51(45.5%)	61 (45.2%)	0.202	0.653
Popliteal artery	19(17%)	23(17%)	0.010	0.921
Tibial artery	16(14.3%)	19(14.1%)	0.023	0.879
**Clinical symptom**				
claudication	14 (22.6%)	12(15.6%)	1.106	0.293
rest pain	25 (40.3%)	33(42.9%)	0.091	0.763
gangrene or tissue loss	23 (37.1%)	32(41.5%)	0.286	0.593

*Abbreviations*: ABI, ankle brachial indexes

The mean values of ABI were significantly increased after primary intervention (0.397 ± 0.125 versus 0.779 ± 0.137, t = -25.780, *P *< 0.001) in diabetes group and (0.406 ± 0.101 versus 0.786 ± 0.121, t = -37.221, *P *< 0.001) in non-diabetes group.

**Figure 1:**
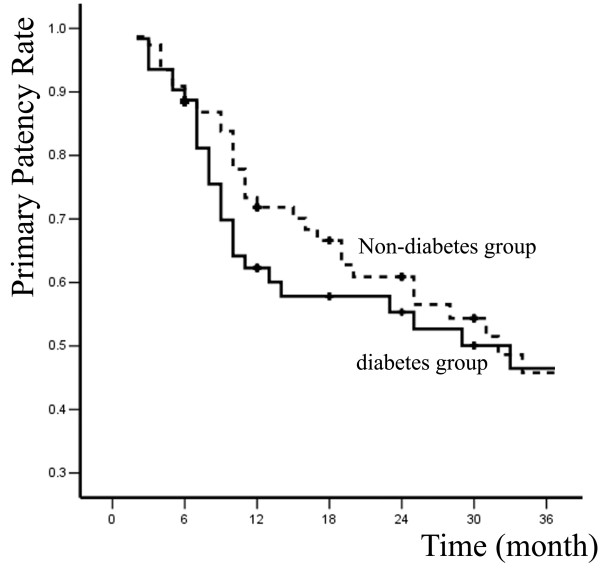
**Primary patency rates of two groups**.

**Figure 2:**
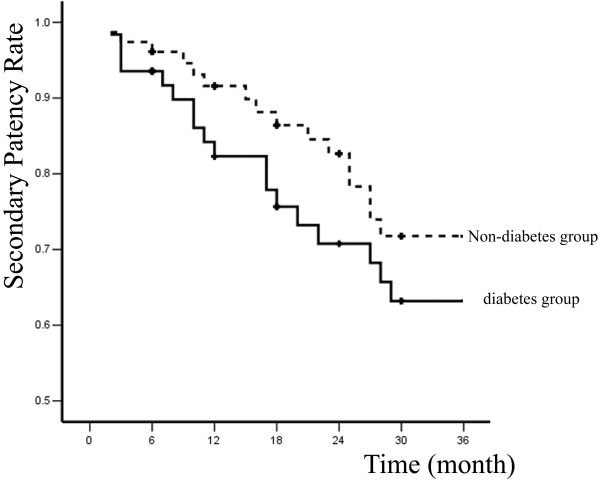
**Secondary patency rates of two groups**.

**Figure 3:**
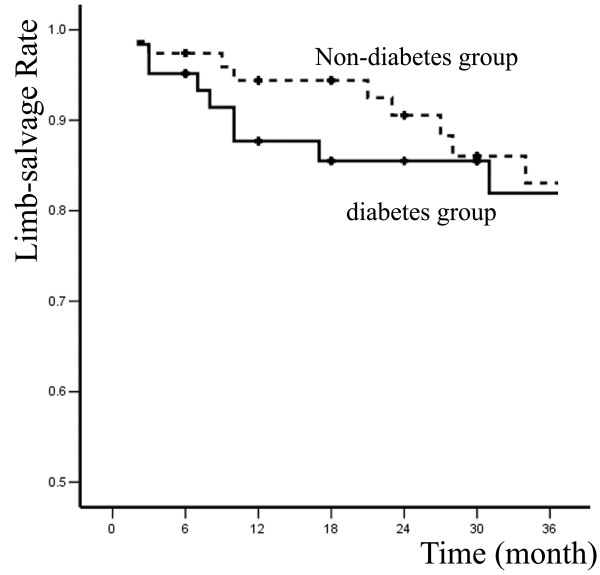
**Limb-salvage rates of two groups**.

Primary and secondary patency rates for all interventions and limb-salvage rates for patients are illustrated by Kaplan-Meier curves (Figures 1, 2 and 3 and Table 2).

**Table 2:** The results of primary, secondary patency rates and limb-salvage rates for all patients

		6 months	12 months	24 months	36 months
Primary	Diabetic (n = 62)	88.7% ± 4.0%	62.3% ± 6.6%	55.3% ± 7.0%	46.5% ± 7.5%
patency rate	Non-diabetic (n = 77)	90.9% ± 3.3%	71.8% ± 5.4%	71.8% ± 5.4%	60.9% ± 6.2%
Secondary	Diabetic (n = 62)	93.5% ± 3.1%	82.3% ± 5.1%	70.8% ± 6.5%	65.7% ± 7.0%
patency rate	Non-diabetic (n = 77)	96.1% ± 2.2%	91.6% ± 3.3%	82.7% ± 4.8%	71.8% ± 6.2%
Limb-salvage	Diabetic (n = 62)	95.2% ± 2.7%	87.7% ± 4.4%	85.5% ± 4.8%	81.9% ± 5.8%
rate	Non-diabetic (n = 77)	97.4% ± 1.8%	94.4% ± 2.7%	90.6% ± 3.7%	83.1% ± 5.4%

In diabetes group, 6, 12, 24, and 36-month primary patency rates were 88.7% ± 4.0%, 62.3% ± 6.6%, 55.3% ± 7.0%, and 46.5% ± 7.5%; whereas secondary patency rates were 93.5% ± 3.1%, 82.3% ± 5.1%, 70.8% ± 6.5%, and 65.7% ± 7%; limb-salvage rates were 95.2% ± 2.7%, 87.7% ± 4.4%, 85.5% ± 4.8%, and 81.9% ± 5.8%. In non-diabetes group, 6, 12, 24, and 36-month primary patency rates were 90.9% ± 3.3%, 71.8% ± 5.4%, 71.8% ± 5.4%, and 60.9% ± 6.2%; secondary patency rates were 96.1% ± 2.2%, 91.6% ± 3.3%, 82.7% ± 4.8%, and 71.8% ± 6.2%; limb-salvage rates were 97.4% ± 1.8%, 94.4% ± 2.7%, 90.6% ± 3.7%, and 83.1% ± 5.4%. The differences between two groups were not significant (*P *> 0.05).

**Figure 4:**
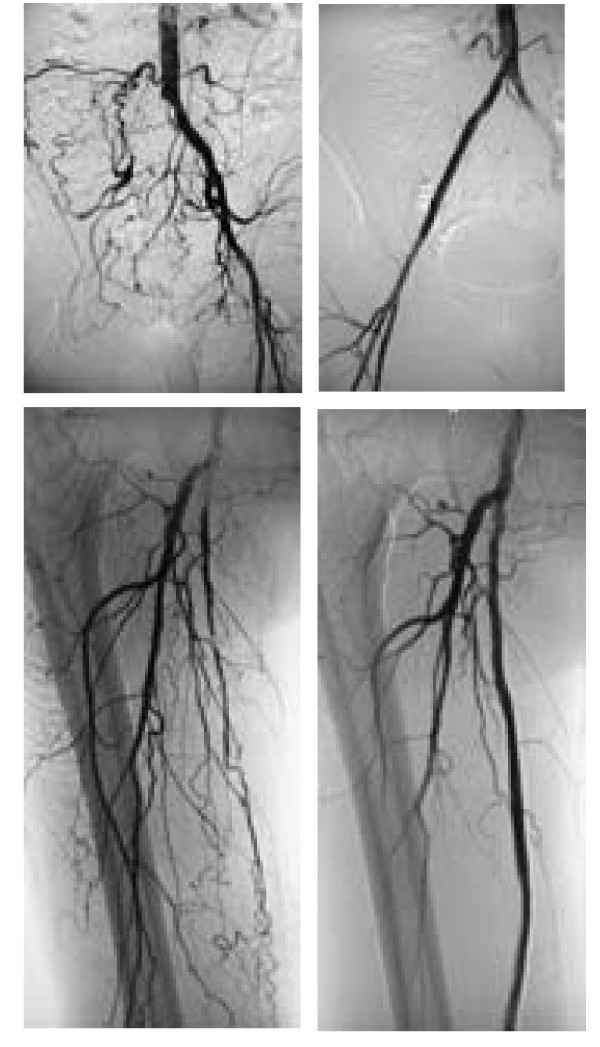
**Iliaco-femoral arterial long occlusion. (a) **DSA displayed right iliac artery overall occlusion; **(b) **after PTA and stenting, blood flow in right iliac artery was recovered; **(c) **DSA displayed right superficial femoral artery overall occlusion; **(d) **after PTA and stenting, blood flow in right superficial femoral artery was recovered.

**Figure 5:**
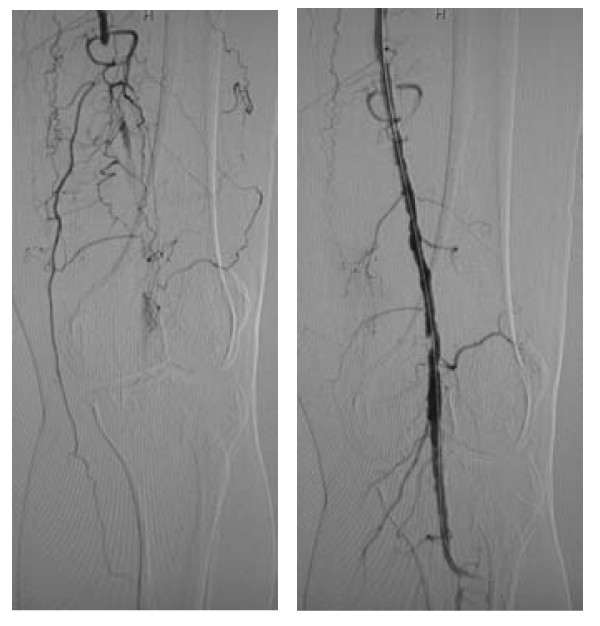
**Popliteal arterial long occlusion case. (a) **DSA displayed left popliteal artery overall occlusion; **(b) **after PTA and stenting, blood flow in left popliteal artery was recovered.

**Figure 6:**
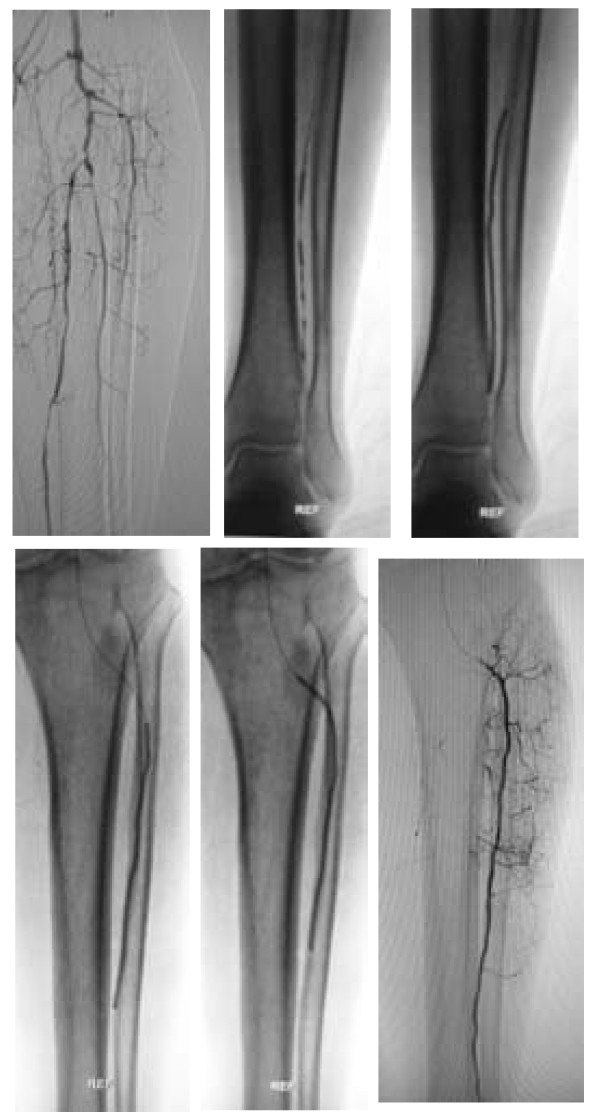
**Anterior tibial artery long occlusion case. (a) **DSA displayed left anterior tibial artery long occlusion; **(b-e) **Deep balloon (2.5 mm × 80 mm) dilated the anterior tibial artery; **(f) **after PTA, blood flow in left anterior tibial artery was recovered.

**Figure 7:**
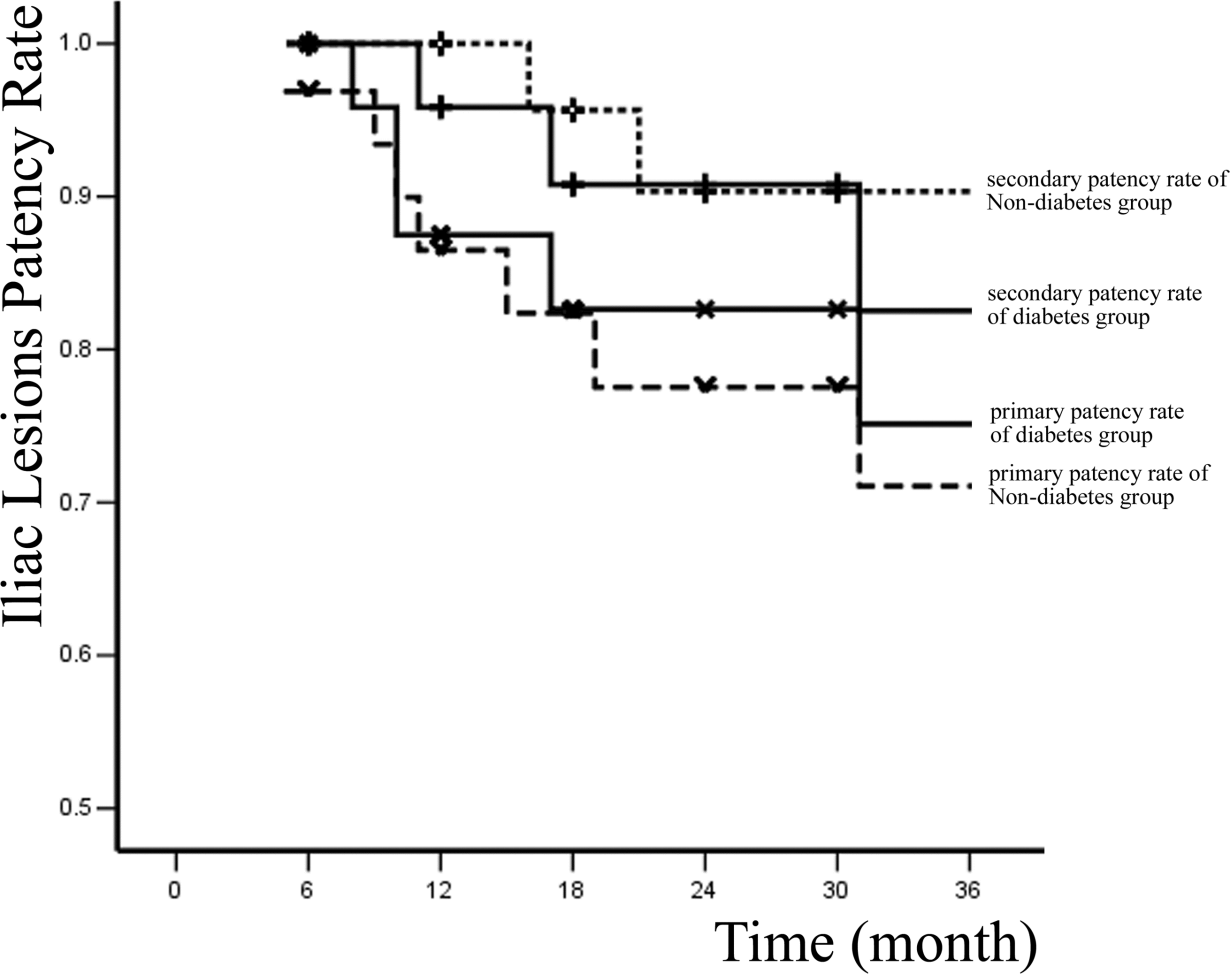
**Primary, secondary patency rates for iliac lesions in two groups**.

**Figure 8:**
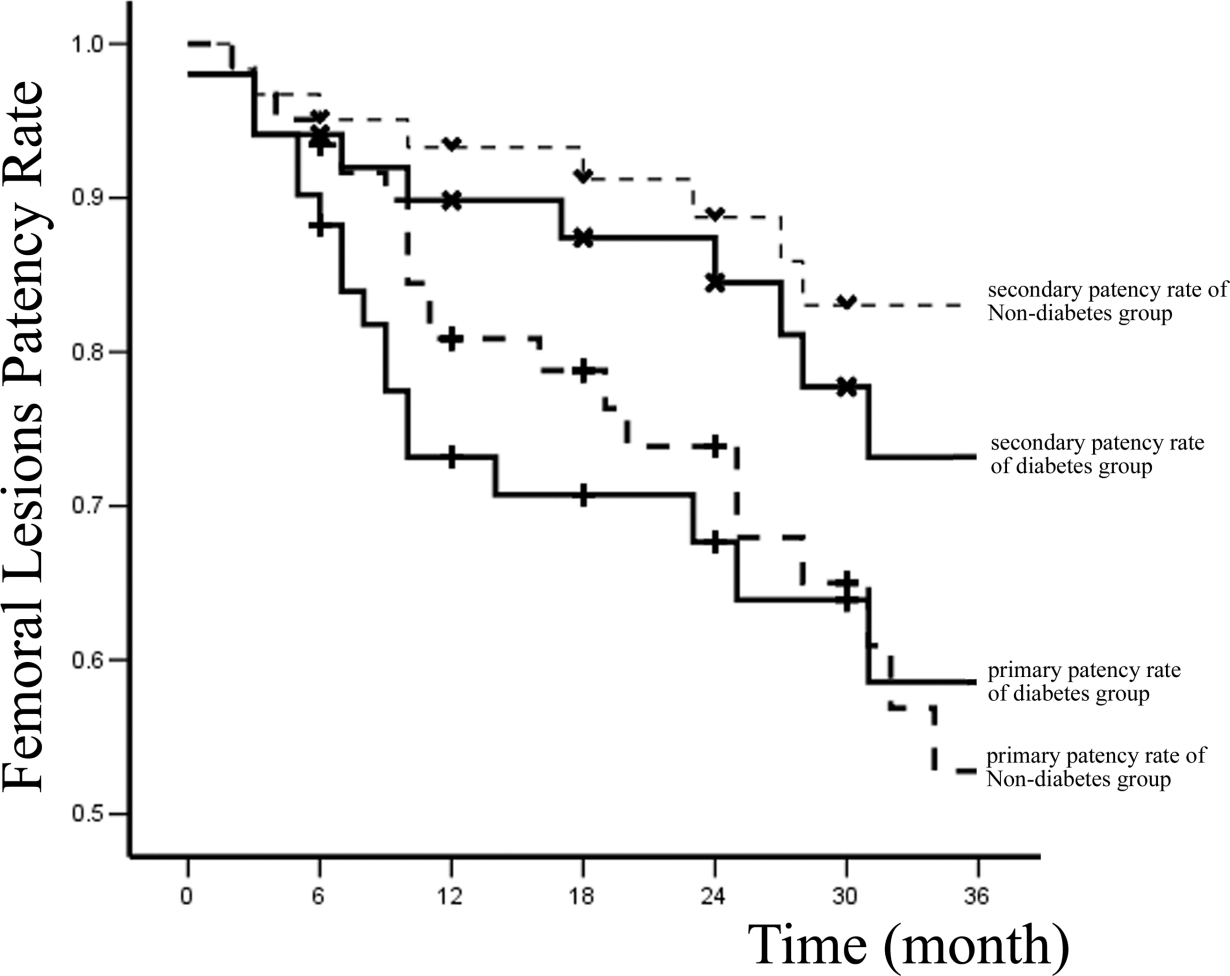
**Primary, secondary patency rates for femoral lesions in two groups**.

**Figure 9:**
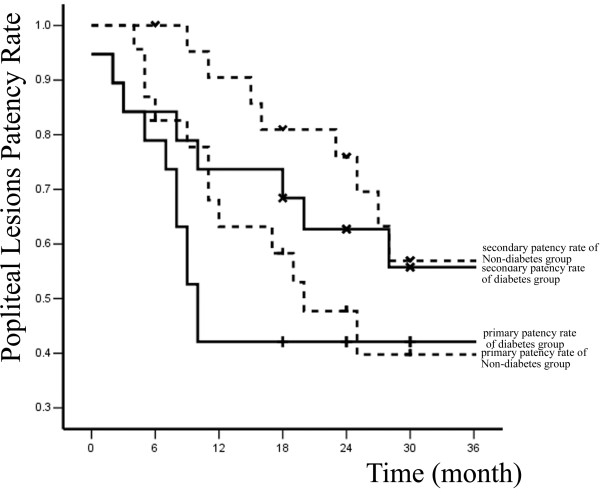
**Primary, secondary patency rates for popliteal lesions in two groups**.

**Figure 10:**
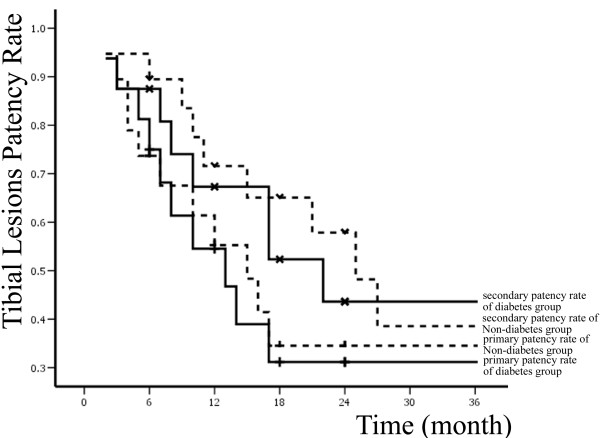
**Primary, secondary patency rates for tibial lesions in two groups**.

A total of 83 percutaneous interventions (32 PTA only, 51 PTA and Stent) included 62 primary interventions and 21 re-interventions were performed on 62 diabetic patients. 103 percutaneous interventions (39 PTA only, 64 PTA and Stent) included 77 primary interventions and 26 re-interventions were performed on 77 non-diabetic patients. All lesions were categorized by location: 58 were iliac lesions (23.5%)(Figure 4), 112 were femoral lesions (45.3%)(Figure 4), 42 were popliteal lesions (17.0%)(Figure 5) and 35 were tibial lesions (14.2%)(Figure 6). 6, 12, 24, and 36-month primary and secondary patency rates for different lesions in diabetes group and non-diabetes group are illustrated by Kaplan-Meier curves (Figures 7, 8, 9 and 10 and Tables 3 and 4).

**Table 3:** The results of primary and secondary patency rates for different lesions in diabetes group

		6 months	12 months	24 months	36 months
iliac lesions (n = 26)	primary patency rate	100%	87.5% ± 6.8%	82.6% ± 7.9%	75.1% ± 10.2%
	secondary patency rate	100%	95.8% ± 4.1%	90.8% ± 6.2%	82.5% ± 9.7%
femoral	primary patency rate	88.2% ± 4.5%	73.2% ± 6.4%	67.7% ± 7%	58.6% ± 8.6%
lesions (n = 51)	secondary patency rate	94.1% ± 3.3%	89.8% ± 4.3%	84.5% ± 5.5%	73.2% ± 7.8%
popliteal	primary patency rate	78.9% ± 9.4%	42.1% ± 11.3%	42.1% ± 11.3%	42.1% ± 11.3%
lesions (n = 19)	secondary patency rate	84.2% ± 8.4%	73.7% ± 10.1%	62.7% ± 11.2%	55.8% ± 11.9%
tibial	primary patency rate	75.0% ± 10.8%	53.8% ± 12.9%	30.8% ± 12.5%	30.8% ± 12.5%
lesions (n = 16)	secondary patency rate	87.5% ± 8.3%	67.3% ± 12%	43.6% ± 13.6%	43.6% ± 13.6%

**Table 4:** The results of primary and secondary patency rates for different lesions in non-diabetes group

		6 months	12 months	24 months	36 months
iliac lesions (n = 32)	primary patency rate	96.9% ± 3.1%	86.5% ± 6.3%	77.5% ± 8.3%	71.1% ± 9.8%
	secondary patency rate	100% ± 0%	100% ± 0%	90.3% ± 6.5%	83% ± 5.7%
femoral lesions	primary patency rate	93.4% ± 3.2%	80.9% ± 5.2%	73.9% ± 6.1%	52.8% ± 8.6%
(n = 61)	secondary patency rate	95.1% ± 2.8%	93.3% ± 3.2%	88.7% ± 4.4%	83% ± 5.7%
popliteal	primary patency rate	82.6% ± 7.9%	63.2% ± 10.4%	47.7% ± 11.1%	39.8% ± 11.7%
lesions (n = 23)	secondary patency rate	100% ± 0%	90.5% ± 6.4%	75.9% ± 9.4%	56.9% ± 11.8%
tibial	primary patency rate	73.7% ± 10.1%	55.3% ± 11.9%	34.5% ± 12%	34.5% ± 12%
lesions (n = 19)	secondary patency rate	89.5% ± 7%	71.6% ± 10.8%	57.8% ± 12.4%	38.6% ± 13.9%

Interventional operation success rate in diabetes group is 98.4% (61/62). In one patient with PAOD (1.6%), there was failure to cross the total occlusion with a wire. The patient's condition was not worsened by the attempted intervention. The patient had successful distal bypass. Interventional operation success rate in non-diabetes group is 100% (77/77).

Perioperative 30-day mortality was 0%. Major complications were access-related and included groin hematoma in 7.2% patients (all not requiring operative exploration), and pseudoaneurysm formation in 2.2% patients (successfully treated by local pressure bandaging).

Mean follow-up time for all patients was 26.3 ± 15.2 months (6-48 months). 47 patients accepted re-interventional operation after restenosis, with success rate 80.9% (38/47). The overall rate of percutaneous re-intervention during the follow-up period was 33.8% (47/139). After failure of percutaneous therapy or restenosis, 21 patients with limb-threat underwent surgical bypass for limb-salvage with success rate 76.2% (16/21), and 19 patients underwent lower extremity major amputation.

## Discussion

### Etiology and prognosis

The hyperglycemia and lipid metabolic abnormality not only lead to injury of the vascular endothelial cells, but also initiate and promote the process of atherosclerosis in diabetes. Some protective cytokines, such as adiponectin and osteoprotegerin, could relieve the progression of PAOD. Adiponectin exhibits anti-inflammatory and atheroprotective actions and osteoprotegerin protects vascular endothelial cells and inhibits atherosclerosis. Lower plasma levels of adiponectin and higher osteoprotegerin were found in diabetic patients with PAOD [9-11]. In fact, the incidence of atherosclerosis in diabetes was significantly higher than that in non-diabetic patients, and the lesion in diabetes was more serious and diffuse. For diabetes, atherosclerosis usually involved the below-knee arteries; the severe vascular lesions and their wide range often lead to less effective intervention; on the other hand, initial hyperplasia or smooth muscle cell proliferation and the progression of atherosclerosis was more serious in diabetes than that in non-diabetes after revascularization.

Patients with PAOD have a poor clinical course. During the first year of diagnosis, mortality is 20%-30%, and an equivalent number undergo amputations or suffer from persistent pain [12]. Lower extremity bypass has been applied very successfully even in difficult clinical cases, including the treatment of patients with diabetic foot gangrene and other conditions which carry a high risk of amputation [13]. But bypass surgery can only be performed to a minority of patients because of poor runoff, advanced age, and cardiac co-morbidities. Surgical treatment is often withheld from those patients who would benefit. Surgical complications include death (1.3%-6%), myocardial infarction (1.9%-3.4%), wound infections (10%-30%), leg edema (50%-100%), and early graft failure rates (6%-49%) requiring repeat surgery [12,14].

### Therapeutic regimen

Angioplasty for critical limb ischemia was reported nearly 50 years ago [15]. Hanna et al. [16] reported a 21% procedural complication rate, including embolization and thrombosis in patients with critical limb ischemia undergoing balloon angioplasty, whereas Dorros et al. [17] demonstrated that PTA of below-knee arteries could be performed with relative safety and with satisfactory results.

The mortality and major morbidity in patients treated for limb-threatening disease with percutaneous interventions was only 0 and 8.1%, respectively, compared with usual mortalities and morbidities of 5% and 30% for open surgical bypass [12]. To some extent, it appears that the short-term advantages of minimally invasive vascular therapies are often counterbalanced by diminished durability. Percutaneous therapy for limb-threatening lower extremity vascular disease with or without diabetes has no exception, with primary patency rates at 36 months of 46.5% and 60.9%. However, secondary patency rates were more reasonable at 65.7% and 71.8%, and more importantly, limb salvages rates were 81.9% and 83.1% at 36 months.

### Efficacy of different studies

It was confirmed that PTA was an effective and safe therapy for short iliac arterial stenosis. But the long-term patency rates of PTA in complicated iliac arterial lesions such as long occlusion were relative low. The 3-year primary patency rates in PTA group were lower than 60% [18,19]; whereas primary patency rates in stenting group were up to 90% [20-22]. Bosch and Hunink [23] reported the technical success rate of interventional therapy for iliac occlusive disease in stent group was higher than that in PTA group, but the difference was not statistically significant. In our study, 1-year and 3-year primary patency rate for iliac arterial lesions in diabetes group or non-diabetes group were 87.5%, 75.1% and 86.5%, 71.1%, respectively.

Bakken et al. [24] reported the clinical results of interventional therapy for femoral lesions with diabetes was better than that without diabetes. In our study, 1, 3 years primary patency rate for femoral lesions in with or without diabetes group were similar (73.2%, 58.6% and 80.9%, 52.8%). Role of femoral arterial stents in the management of PAOD was still controversial. In one meta-analysis [25] which included 452 patients who underwent femoropopliteal PTA and 482 patients who underwent femoropopliteal stenting, the 1-year primary patency rates following PTA ranged from 45% to 84.2% and at 2 years it varied from 25% to 77.2%, the 1-year primary patency rates in the stent implantation group varing from 63% to 90%, and 2-year primary patency ranging from 46% to 87%. In 73 patients with average 8 cm long femoral arterial stenosis or occlusion, restenosis rates in the stent and PTA groups were 2.9% versus 18.9%, 18.2% versus 50.0%, and 34.4% versus 61.1% at 3, 6, and 12 months by sonography [26]. Perrio et al. [27] reported 1 year femoral artery primary patency rates were 57% in stent group and 53% in PTA group. Kougias et al. [28] reported at 1 year femoral artery primary patency for the subintimal balloon angioplasty and subintimal placement of covered stent groups was 28% versus 75%, whereas secondary patency was 37% versus 84%. But in other study undergoing subintimal angioplasty in the femoral and popliteal arteries, one-year primary and secondary patency for stent vs. no-stent group was 50% versus 45% and 70% versus 78% [29]. In a contrast study between selective stenting and systematic stenting of femoral artery, Becquemin et al. [30] reported at 1-year follow-up restenosis of the treated site was noted in 32.3% patients in selective stent group and 34.7% patients in systematic stent group. After 4 years, limb-salvage rates in selective stent group and in systematic stent group were 57%, 44%, respectively. Systematic stenting in femoral artery did not improve long term patency.

The treatment of infra-popliteal arterial occlusive disease was still a clinical puzzle for vascular surgeons and interventional radiologists. The first reason was that infra-popliteal arterial occlusive disease usually comorbided with diabetic arteriosclerosis which have more calcification and more hard plaque. The second reason was that the outflow tract of infra-popliteal artery has higher resistance. In a meta analysis, Romiti et al. [31] reported the immediate technique success rate of PTA in infra-popliteal arterial occlusive disease was 89%. After 12 and 36 months primary patency rates, secondary patency rates, and limb salvage rates were 77.4% and 48.6%, 83.3% and 62.9%, 93.4% and 82.4%, respectively. Lejay et al. [32] reported at 1 year the infra-popliteal arteries primary patency rates and limb salvage rates for PTA were 60% and 85%, respectively. In a contrast study between PTA and atherectomy of infra-popliteal arterial occlusive disease, Semaan et al. [33] reported primary patency of the popliteal artery at 12 months were 75% and 73%, respectively, but the difference was not statistically significant. In our study, 6, 12 months primary patency rate for tibial arterial lesions with or without diabetes were 75%, 53.8% and 73.7%, 55.3%. The result was lower than the result in literature. 26 patients (26/35) in the study comorbided with proximal arterial occlusive lesions may be the origin.

### Limitations

This was a single-center, consecutive patient trial. ABIs were obtained before intervention to evaluate hemodynamic impairment and at one week after intervention to measure improvement. ABI value responded objectively to the status of blood supply in lower extremity, but the mickle factors influenced its magnitude. In the condition of profuse collateral circulation in lower extremity, ABI value could exactly respond to the extent of vascular flow in treated position. The sample number of this study is relative deficient, and subgroups divided by distinct lesions position had not enough information for contrasting with literature. As the follow-up period was relative short, this study could not illuminate the long term patency of interventional therapy for PAOD.

## Conclusion

Percutaneous revascularization has rapidly emerged as an alternative to open surgical bypass for patients with chronic lower extremity ischemia. In this study, the good short-term patency rate of percutaneous intervention makes 81.9% diabetic PAOD patients and 83.1% non-diabetic PAOD patients keep the limb within three years. With a low risk of morbidity and mortality, the percutaneous revascularization accepted by patients does not affect ultimate necessary surgical revascularization and consequently should be considered as the preferred therapy for chronic lower extremity ischemia. The efficacy and prognosis of interventional therapy in diabetic patients is similar that in non-diabetic patients.

## Abbreviations

PAOD: Peripheral arterial occlusive disease; ABI: Ankle brachial indexes; 3D-CTA: Three dimensional computed tomography angiography; PTA: Percutaneous transluminal angioplasty; SPSS: Statistical Package for Social Sciences.

## Competing interests

This article was supported by research grants from the Scientific Research Fund of Liaoning Science and Technology Agency, China (No. 2008225010-5) and the Scientific Research Fund of Liaoning Education Agency, China (No. 2007T183) and the Scientific Research Fund of First Hospital of CMU (No. FSFH1006).

The authors have not signed an agreement with any sponsor of the study reported in this article that has a clause which prevents us from publishing both positive and negative results, from collaborating with other investigators to pool data across sites, or that forbids us from publishing without the approval of the sponsor.

The authors declare that they have no competing interests.

## Authors' contributions

LX conceived of the study, and participated in its design and coordination, carried out the clinical studies and helped to draft the manuscript. D-sH participated in the design of the study and performed the statistical analysis. J-jT carried out the clinical studies and drafted the manuscript. JS carried out the clinical studies and drafted the manuscript. All authors read and approved the final manuscript.
